# Mediterranean isolation preconditioning the Earth System for late Miocene climate cooling

**DOI:** 10.1038/s41598-019-40208-2

**Published:** 2019-03-07

**Authors:** Walter Capella, Rachel Flecker, F. Javier Hernández-Molina, Dirk Simon, Paul Th. Meijer, Mike Rogerson, Francisco J. Sierro, Wout Krijgsman

**Affiliations:** 1 Department of Earth Sciences, Utrecht University, Utrecht, The Netherlands; 2 Department of Earth Sciences, Royal Holloway, University of London, Egham, UK; 3 BRIDGE, School of Geographical Sciences and Cabot Institute, University of Bristol, Bristol, UK; 4 Geography Department, University of Hull, Hull, UK; 5 Department of Geology, University of Salamanca, Salamanca, Spain

## Abstract

A global Neogene cooling trend culminated ~7 million years ago with the onset of Greenland glaciation. Increased ocean-atmosphere interaction and low- to high-latitude circulation are thought to be key factors in reorganizing late Miocene global temperature and precipitation patterns, but the drivers of this reorganization have yet to be identified. Here, we present new information about the evolution of the Atlantic-Mediterranean gateway that generated Mediterranean overflow. We use sedimentary and palaeogeographic evidence to constrain the timing and dimensions of this gateway and document the initiation of a saline plume of water within the North Atlantic. Today, this saline jet entrains and transports Eastern North Atlantic water and its dissolved inorganic carbon into the interior of the ocean, contributing to the drawdown of CO_2_ and the sensitivity of the ocean to atmospheric changes. We show that during the Miocene this transport emerged simultaneously with gateway restriction and propose that the resulting interaction of ocean-surface and ocean-interior carbon inventories would have greatly enhanced ocean-atmosphere exchange, preconditioning the Earth System for late Miocene cooling.

## Introduction

**Figure 1:**
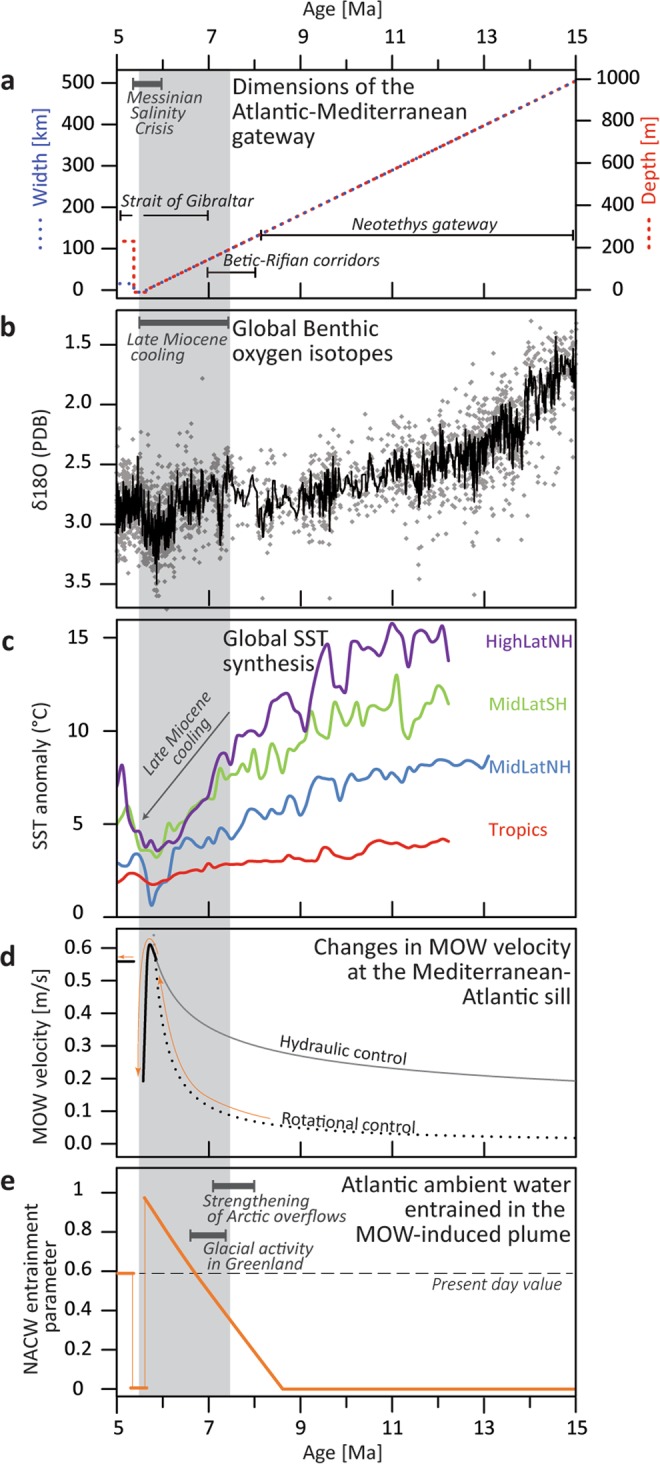
Middle to late Miocene climate and Atlantic-Mediterranean gateway changes. (**a**) Approximate time trend of the gateway width and depth throughout the late Miocene including the combined dimensions of existing gateways for a given time (reconstructed linearly from palaeogeographic constraints, Fig. 2). (**b**) Benthic δ^18^O composite^3^; PDB, PeeDee Belemnite. (**c**) Sea surface temperatures for Northern Hemisphere high- and mid-latitudes, Southern Hemisphere mid-latitudes, and Tropics^1^. Grey shaded area represents the duration of late Miocene surface water cooling^1^. (**d**) Velocity of Mediterranean Overflow Water (MOW) with varying dimensions of the Atlantic-Mediterranean gateways (**a**) computed with rotational- and hydraulic-control theories^53^. Bold lines indicate the preferred theory to compute velocity for a given state of the gateway: rotational control theory is more accurate for larger gateways, whereas hydraulic control becomes preferable once the two lines intersect. (**e**) Proportion of the water from the upper layers of the North Atlantic gyre (NACW) that is entrained into the MOW-generated plume, based on the calculations and references shown in Supplementary Table 1 (Supplementary Material). Evidence of strengthening arctic overflows are based on refs^60,61^. Pockets of glacial activity in Greenland are from refs^62,63^.

A pronounced phase of global palaeogeographic, biologic, and climatic turnover occurred during the late Miocene^1,2^ with sea temperatures showing different spatial patterns in the deep (Fig. 1b) and shallow realms (Fig. 1c). Significant deep-sea water cooling occurred around 12–15 Ma, after the Middle Miocene Climatic Optimum^3^ and then remained rather constant during the late Miocene (Fig. 1b). In contrast, up to 6 °C of sea surface temperature (SST) cooling occurred between ~7.5 and 5.5 Ma^1^ (Fig. 1c). This cooling trend is visible in both hemispheres and across the world’s major oceans. It amplifies towards the high latitudes, and terminates with temperatures almost as low as modern day values^1^. This enigmatic, late Miocene global climatic turnover has been attributed to two separate hypotheses. The first^1^ is based on the response of the coccolithophorid algae vital-effect^4^ and suggests that a decline in CO_2_ could have occurred, albeit in contrast to values of CO_2_ recorded by other proxies that show no significant change^1,5^. The second is based on stable isotope data showing ocean – atmospheric CO_2_ decoupling during the late Miocene^6^, and suggests that changes in ocean gateways created a relatively deep global thermocline and reductions in low-latitude gradients in sea surface temperature. Here, we explore the impact of restricting the Atlantic-Mediterranean gateway and initiating Mediterranean overflow. This late Miocene oceanographic change may have had far-reaching implications, not just for sea-surface temperature changes, but also for CO_2_ exchange between the ocean and atmosphere.

The ocean is the largest of the rapidly exchanging CO_2_ reservoirs, and thermohaline circulation can modify the sensitivity of the ocean to atmospheric carbon changes via opening and closing of key ocean gateways^6–8^. In palaeoclimate models, parameterization of exchange through ocean gateways is difficult because of their small spatial scale relative to the size of model grid cells^9,10^. Generally, coeval geological records reflecting gateway configuration changes are used as the basis for largely qualitative assumptions about the drivers^11^. Here, we present new sedimentary and palaeogeographic data from the late Miocene Atlantic-Mediterranean gateway^12,13^ that provide key information on the gateway’s behaviour through time. Combining these observational constraints with strait dynamic theory^14,15^, we quantify first-order variations in late Miocene Atlantic-Mediterranean exchange, its velocity and ability to entrain and transport surface waters. We then explore the implications of increased interaction between the lower and upper parts of the ocean at a time of global cooling.

## Atlantic-Mediterranean exchange, properties and processes

In the Atlantic, several marine overflows (Denmark Strait, Mediterranean, Weddell Sea) supply the dense water that collectively feeds the thermohaline circulation system^16^. Of these, the transportation of hypersaline water from the Mediterranean into the interior of the Atlantic is amongst the largest and densest in the global ocean^9^ and the exchange also provides a key exit point for Atlantic buoyancy, which is the underlying driver behind Atlantic deep convection^17^.

The Mediterranean’s dense outflow is generated as a consequence of its mid-latitude setting where evaporation exceeds precipitation^18^ forming a warm, but salty water mass. This negative hydrologic budget amplifies the climate signal transmitted principally through the Mediterranean’s southern catchments from North African monsoon rainfall^19^. The sub-tropical monsoonal climate signal with its strong precessional pulse, is then propagated into the Atlantic by density-driven exchange^20^ through the Gibraltar Strait. Water flowing out of the Mediterranean at depth entrains ambient Atlantic water as it goes^21^, generating Atlantic-Mediterranean Water^22^. This distinctive water mass forms large depositional and erosional features including extensive sandy contouritic drifts^23^, which allow the reconstruction of Mediterranean overflow activity in the past^24,25^. Atlantic-Mediterranean Water flows north, fuelling the Norwegian Seas with higher density water that helps to sustain the formation and southward flow of North Atlantic Deep Water^22^.

Despite the challenges of modelling the gateway, the exchange that occurs through the Gibraltar Strait today is a sufficiently influential component of the Earth System for general circulation models to capture at least part of its impact^26,27^. Experiments without Atlantic-Mediterranean exchange show that its presence makes Greenland warmer and Antarctica cooler^27^. This in turn, is sufficient to shift the position of the Intertropical Convergence Zone, and hence the location of monsoons, storm tracks and the hyper-arid zones between them. Atlantic-Mediterranean exchange is also a critical component of Atlantic Meridional Overturning Circulation (AMOC) particularly at times of weak North Atlantic Deep Water formation^26,28–32^, as a consequence of dense, salty water being transported from Gibraltar into the high latitudes. Given the weaker AMOC during the late Miocene^33,34^, the relative contribution of Mediterranean density to late Miocene deep-water formation is likely to have been greater than today^10^. Furthermore, in entraining ambient Atlantic water, the transport of dense Mediterranean Overflow into the Atlantic interior also transfers 0.06 GtC yr^1^ of anthropogenic carbon from the ocean surface to intermediate depths^35^, contributing a 2–5% of today’s total net ocean carbon sink^36–38^.

**Figure 2:**
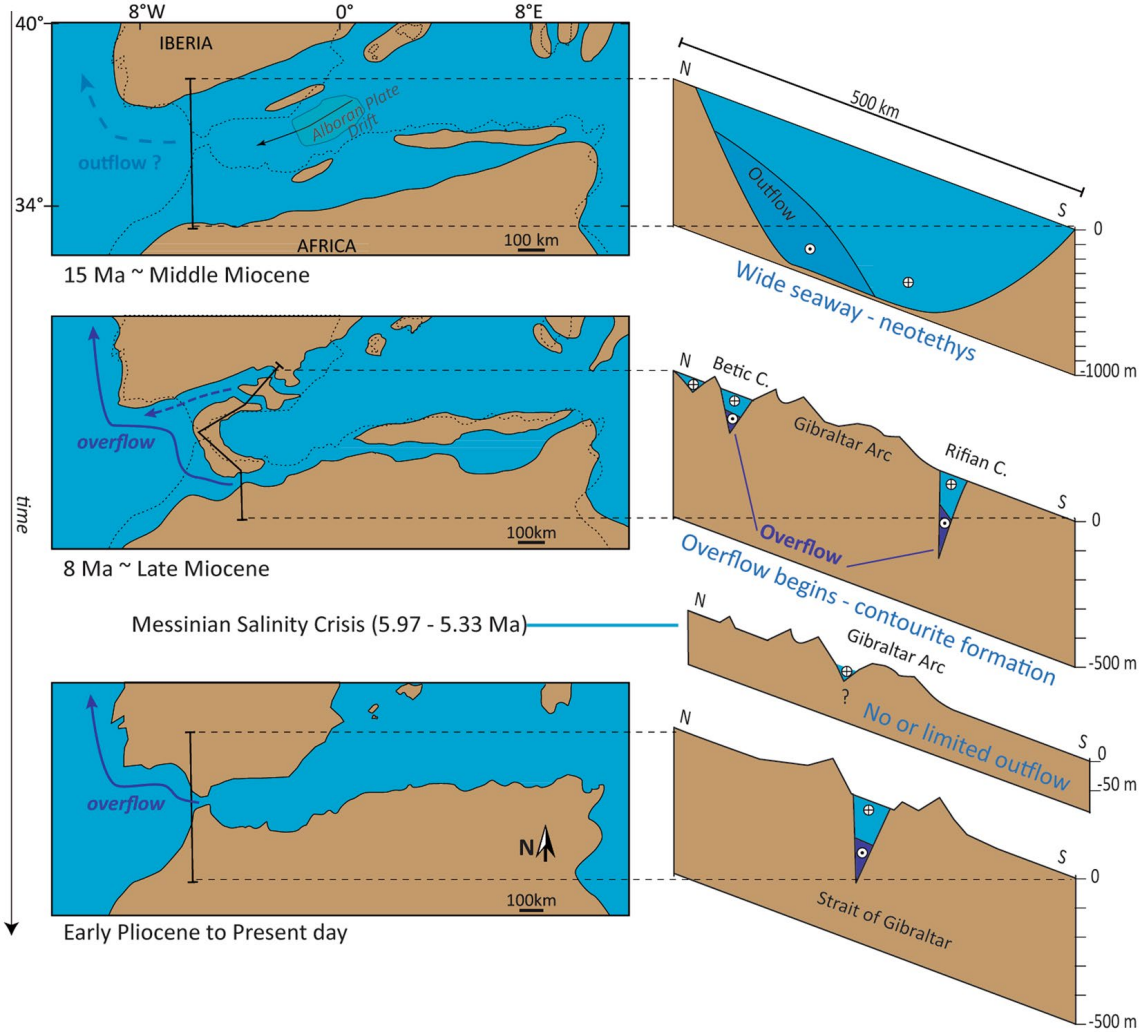
Three-step sketch showing the tectonically-controlled reconfiguration of the Atlantic-Mediterranean gateways from middle Miocene to present-day. Paleogeography of the Western Mediterranean after^12^. The black arrow in the middle Miocene configuration depicts the approximate path of the Alboran Plate drift, that occurred between the early-middle Miocene^64^. The formation of the Gibraltar arc created the Rifian/Betic corridors and replaced a wider gateway in which Mediterranean outflow distribution was influenced only by rotational control. The late Miocene scenario is the first with hydraulic control on flow (Fig. 1d) and potential impact on Atlantic-Mediterranean salinity gradients and overflow formation.

Exchange through Gibraltar, however, is a relatively recent phenomenon (Fig. 2). Progressive isolation of the Mediterranean from the Atlantic occurred throughout the Miocene, driven by Africa-Eurasia convergence coupled with the westward drift of the Alboran Plate^39^. This restriction replaced a wide gateway (Fig. 2) floored with oceanic crust^40^ with narrower, shallower connections: the Rifian and Betic corridors^41,42^, through which Atlantic-Mediterranean exchange was funnelled (Fig. 2).

The onset of episodic organic-rich sedimentation (sapropels) in the Middle Miocene^43^ is the earliest evidence of Mediterranean oceanography distinct from the Atlantic, although this alone does not require the existence of Mediterranean overflow. Only at some point during the late Miocene did on-going restriction of the marine corridors permit Mediterranean salinity to rise forming a dense water mass that overspilled into the Atlantic for the first time^13^. Progressive narrowing and closure of these connections resulted in extreme salinity fluctuations in the Mediterranean (Fig. 2), leading to the precipitation of more than 1 million km^3^ of salt, equivalent to ~6% of the total dissolved oceanic NaCl in the latest Miocene ocean^44,45^. This event, known as the Messinian Salinity Crisis, lasted between 5.97–5.33 Ma^46^. Ultimately, tectonic convergence^39^ coupled with slab-dragging^47^ and isostatic rebound related to slab-tear^48^, not only severed these marine connections, but also uplifted and exposed them on land.

Field evidence from northern Morocco and Southern Spain suggests that the Rifian and Betic Corridor closed in the early Messinian, at about 7 Ma^42,49^ while two-way exchange continued through a proto-Gibraltar Strait^50^.

## New constraints on Late Miocene flow through the Atlantic-Mediterranean gateways

The late Miocene Betic^41^ and Rifian^13^ corridors both contain Miocene contourites (i.e. sediments deposited by bottom-currents) formed by Mediterranean water overflowing into the Atlantic, at flow-velocities greater than 0.5–1 m/s. Here we focus on the Rifian Corridor contourites, which preserve a complete sequence of sediments and sedimentary facies representative of the bottom-current behaviour^13^.

**Figure 3:**
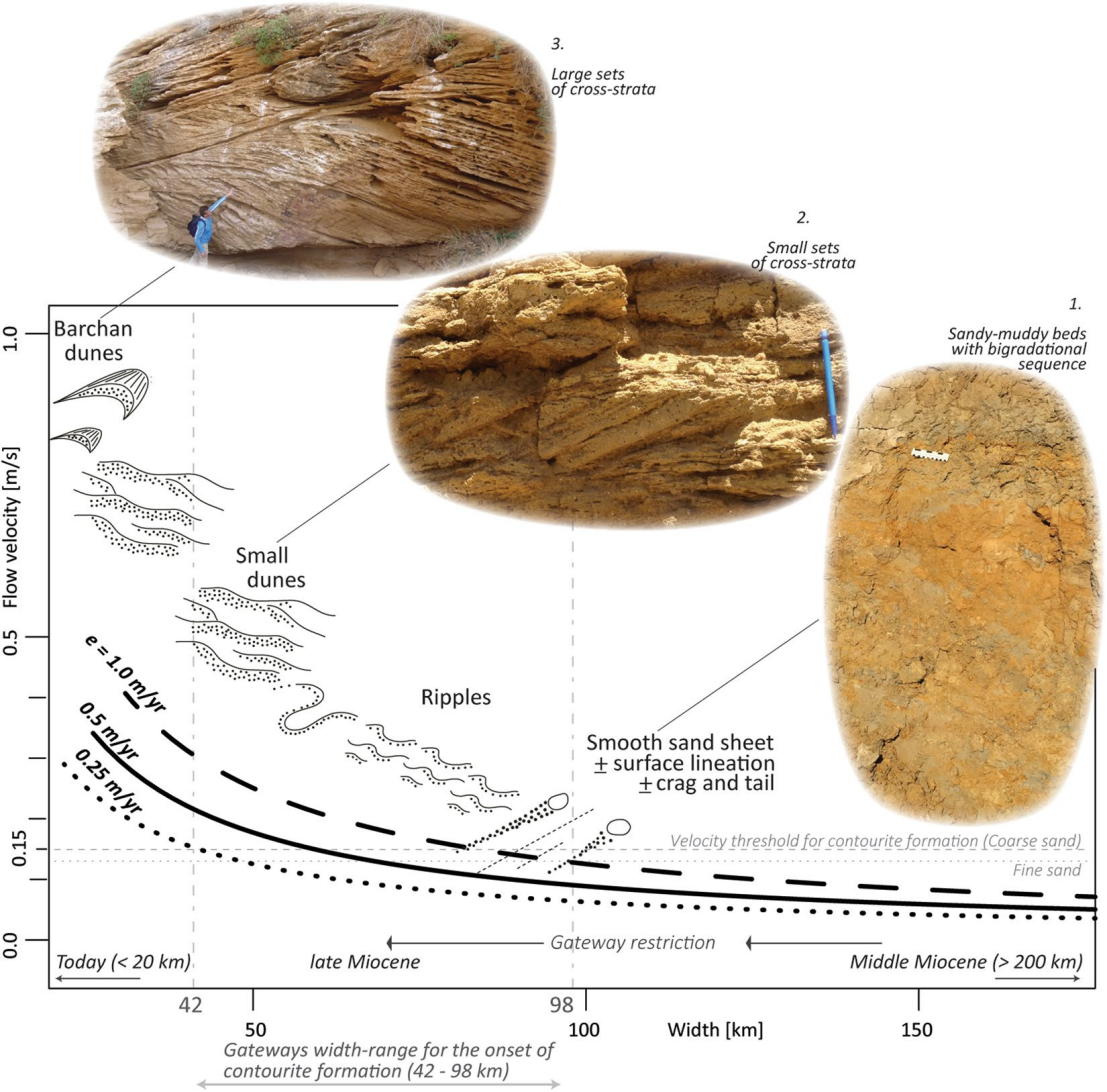
Mean velocity of Mediterranean overflow as a function of gateway width, for three different values of net evaporation over the Mediterranean basin, computed from rotational-control theory^53,54^. High (dashed line) and low (dotted line) evaporation values represent dryer and wetter climatic scenarios, respectively. Note that arid conditions lead to faster overflow for a given width. The black arrows at the bottom show the restriction trend occurring in the Atlantic-Mediterranean gateways through time, as shown in Fig. 2. Different values of grain size lead to different thresholds of flow velocity required to form contourites based on an empirical relationship for deep sea, bottom current-dominated environments^51^. Fine to coarse sand is the dominant grain size of the Rifian Corridor contourites^13^, which are shown in pictures. In picture 1 the scale is 13 cm. See methods for theory details.

Contourites in the Rifian Corridor are composed mainly of bigradational, sandy-muddy beds and cross-stratified sandstone that formed as smooth sand sheets and cross-bedded dunes (Fig. 3). These bedforms are known from deep contourite-dominated environments and for fine to coarse sand represent flow velocities fluctuating between 0.15–1.0 m/s^51^. The occurrence of these sandy contourite bedforms, above non-contouritic mudstone^13^ indicates that bottom-current flow through the Rifian Corridor increased above a critical threshold of 0.13–0.15 m/s^51^.

Field evidence of contourites from the Rifian Corridor allows us to cover the initiation of Mediterranean overflow between 7–8 Ma. Postdating the Betic and Rifian corridors closure at around 7 Ma^42^, we assume that the Strait of Gibraltar took over, accommodating all the exchange^50^. Therefore, to explore the effect of a restricting gateway from its wide oceanic seaway to its modern-like configuration (Fig. 2), we have adopted published paleogeographic and dimensional constraints^12,42,50^, to reconstruct the evolution of a simplified, linearly reducing gateway which narrowed by approximately 500 km and shallowed by about 1000 m during the middle to late Miocene (Fig. 1a).

## The link between sedimentary evidence and overflow behaviour

To find a quantitative link between the field evidence of overflow formation^13^ and the palaeogeographic evolution of the Atlantic-Mediterranean gateway (Figs 1a and 2), we combined the theory of sea straits dynamics with an empirical relationship between flow velocities at the seafloor and the resulting bedforms for a given grain size^51^. The theory (Fig. 3) generates an average overflow velocity as a function of Mediterranean basin evaporation, which in turn influences the water density and thus the vigour of the basin outflow, and of gateway width at the point of greatest constriction, which reflects the process of gradual restriction and approximates the condition and location where the observed contourite bedforms developed (see also methods).

Figure 3 shows the resulting relationship between Atlantic-Mediterranean gateway width (x-axis), which varied during the Miocene (Fig. 2), and the mean velocities of Mediterranean overflow at the sill (y-axis). The model suggests that a reduction in width of the Atlantic-Mediterranean gateway focusses the flow and leads to an increase in overflow velocity. Furthermore, the graph shows three different net evaporation values, covering wetter(dotted line) and dryer (dashed line) periods, representing the possible fluctuations controlled by climate. The theory shows that tectonics is driving the trend towards a narrower gateway, but at any one time, the detail of the regime at the sill itself can be influenced by the Mediterranean climate.

The model results support the notion that only after a certain threshold value of width is passed are velocities high enough to produce erosional and depositional bedforms on the gateway floor, and that the late Miocene, 7–8 Ma gateway reconfiguration (Fig. 2) corresponded to the increase in velocity that deposited coarse and cross-stratified sands in the gateway, corroborating field evidence^13^. Dating the first occurrence of the sandy contourites above finer-grained marlstone^13^ therefore suggests that flow velocity exceeded the critical threshold for contourites formation at around ca. 7.8 Ma and that this is the time at which saline Mediterranean water first started to contribute to North Atlantic circulation.

## Mediterranean overflow velocity and Atlantic water entrainment

The initiation of Atlantic-Mediterranean Water with its characteristic properties and distribution^22^ can therefore be inferred from first sedimentary expressions of restriction in the Betic and Rifian corridors. Since the settling depth of the plume depends predominantly on Atlantic salinity^52^, the effect on surface water entrainment will also have been initiated at this time. The Atlantic-Mediterranean Water plume entrains North Atlantic Central Water (NACW) from within the upper layers of the North Atlantic gyre, taking with it dissolved CO_2_^35^. Increasing overflow velocity leads to an increase in Atlantic entrainment and associated carbon transport^35^.

In Fig. 1e we show the variations of the entrainment parameter^14^, which represents the proportion of NACW entrained by Mediterranean overflow as a function of varying velocity values (Fig. 1d). Velocity values are computed from the dimensions of the Atlantic-Mediterranean gateway at the point of greatest constriction (Fig. 1a) using a simplification for strait exchange^53^ (see methods). NACW becomes entrained within the plume when the gateway restricts beyond a threshold value which here occurred between 9 and 8.5 Ma (Fig. 1e) and increases to a peak value at the beginning of the Messinian Salinity Crisis (Fig. 1a,e). Data from this study therefore suggests that late Miocene restriction of the Mediterranean-Atlantic gateway initiated a new ocean pump that increased the interaction between deep and upper ocean reservoirs^35^ and that this occurred synchronously with the onset of a long-term surface water cooling trend, (Fig. 1c).

## Rapid-moving Mediterranean overflow plume and late Miocene cooling

Global cooling that affected the mid- and high-latitude of both hemispheres between ~7.5 and 5.5 Ma occurred in concert with strengthening of the biological pump^2^ and changes in terrestrial vegetation due to a reduction in late Miocene CO_2_ concentration^1^. We suggest that the synchronous narrowing of the Atlantic-Mediterranean gateway favoured ocean – atmospheric CO_2_ decoupling in two ways: (i) injection of Mediterranean hypersaline waters enhanced northern hemisphere overturning, causing a deepening of the global carbonate compensation depth, shorter residence times of bottom water, and greater sensitivity of the ocean-atmosphere system. At the same time, (ii) by enhanced entrainment of surface water into the Atlantic-Mediterranean Water plume, the new Mediterranean Outflow altered storage of CO_2_ in the Atlantic interior, promoting global changes in the distribution of carbon at the onset of northern hemisphere cooling and during the switch to modern, C_4_-dominated ecosystems.

## Methods

The theory behind Fig. 3 describes the condition of maximal flow in a wide, rotationally controlled gateway with two-way exchange and negligible net flow^54^. For narrower gateways than those considered in this figure, rotational control would give way to hydraulic control^15^, as exemplified in Fig. 1d when width decreases below a certain value. The residence time of the Mediterranean Sea is of the order of 100 s of years, for larger gateway exchange than that presented in this study^55^. However, as we are considering gateway dimension changes on much greater time-scales (Fig. 2), we can assume that the exchange and Mediterranean salinity are close to its equilibrium, allowing us to ignore their time-dependent effect. By having the rotationally-controlled strait connect to a basin subject to a specified net evaporation and ignoring the role of temperature, we can express the flow velocity in terms of gateway dimensions and the value chosen for evaporation. This step assumes that the Rifian corridor exerted the dominant control on basin salinity. While the volume transport of water through the gateway is dependent on strait depth, the mean velocity is not, and therefore we can represent velocity (y axis) as being in function of width (x axis).

How would the results be affected if a significant second gateway was present? If this additional gateway also accommodated outflow then, intuitively, one would expect the outflow through the Rifian corridor to have been less vigorous. This can easily be shown to be consistent with theory as long as the volume transport of the outflow through both gateways is proportional to the salinity (density) excess of the basin. In Fig. 3, a given evaporation and gateway width would thus correspond to lower flow rates than now found. In order to reach the flow velocity required for an observed bedform, a greater restriction would be needed than the one we now infer in the width-range for contourite formation (Fig. 3). The effect of a second gateway is less straightforward in a scenario that it only accommodated inflow. This is expected to happen when the second gateway is relatively shallow^56^. In, perhaps the most likely, situation that the second gateway takes over part of the inflow from the main gateway, the volume transport of outflow would be unaffected but the average outflow velocity may again decrease because the outflow occupies a greater depth range.

Net evaporation is varied around the value of 0.5 m/yr, which is within the correct range for the present-day^57^ and the late Miocene^15^. The velocity at the sill is linked to expected bedform for a grain size of fine (0.125–0.25 mm) to coarse sand (0.5–1 mm) following Stow *et al*.^51^. This range of grain-size reflects the dominant sand composition in the first sandy-muddy beds with bigradational sequences occurring in the Rifian Corridor^13^. In the bedform-velocity matrix of Stow *et al*.^51^ we started with a given grain size (x-axis) and increased flow velocity (y-axis) until we intersected a threshold value representing the minimum velocity to have depositional bedforms on the seafloor (smooth sand sheets and straight ripples).

In the context of the Rifian Corridor, our correlation assumes that the velocity calculated at the sill (i.e. outcome of the computation) and the velocity of the overflow within ~50 km down its path (i.e. where the gateway contourites form) do not differ significantly, as observed in present day monitoring at the exit of the Strait of Gibraltar^58,59^.

The theory behind the entrainment parameter (Fig. 1e) is based on the representation showed in Baringer and Price^14^ and Whitehead^53^ and employs cumulative values of width and depth (Fig. 1a) and resulting velocities (Fig. 1d) for a simplified, linearly-reducing Atlantic-Mediterranean gateway from middle to late Miocene. Supplementary Table 1 (Supplementary Material) shows the calculation of the entrainment parameter for different values of width and depth of the Atlantic-Mediterranean gateway, and resulting MO-velocity. The entrainment parameter indicates the relative proportion of NACW in the Atlantic-Mediterranean Water plume.

## Supplementary information

Supplementary Table 1
